# A Phase II Trial of Adjuvant Durvalumab Following Trimodality Therapy for Locally Advanced Esophageal and Gastroesophageal Junction Adenocarcinoma: A Big Ten Cancer Research Consortium Study

**DOI:** 10.3389/fonc.2021.736620

**Published:** 2021-09-17

**Authors:** Hirva Mamdani, Bryan Schneider, Susan M. Perkins, Heather N. Burney, Pashtoon Murtaza Kasi, Laith I. Abushahin, Thomas Birdas, Kenneth Kesler, Tracy M. Watkins, Sunil S. Badve, Milan Radovich, Shadia I. Jalal

**Affiliations:** ^1^Department of Oncology, Barbara Ann Karmanos Cancer Institute, Wayne State University, Detroit, MI, United States; ^2^Department of Internal Medicine, Division of Hematology Oncology, University of Michigan, Ann Arbor, MI, United States; ^3^Department of Biostatistics and Health Data Science, Indiana University, Indianapolis, IN, United States; ^4^Hematology, Oncology and Bone Marrow Transplantation, Department of Internal Medicine, Holden Comprehensive Cancer Center, University of Iowa, Iowa City, IA, United States; ^5^Department of Internal Medicine, Division of Medical Oncology, The Ohio State University, Columbus, OH, United States; ^6^Department of Surgery, Thoracic Division, Indiana University, Indianapolis, IN, United States; ^7^Department of Pathology, Indiana University, Indianapolis, IN, United States; ^8^Department of Surgery, Indiana University Melvin and Bren Simon Comprehensive Cancer Center, Indianapolis, IN, United States; ^9^Department of Internal Medicine, Division of Hematology/Oncology, Indiana University Melvin and Bren Simon Comprehensive Cancer Center, Indianapolis, IN, United States

**Keywords:** durvalumab, immunotherapy, esophageal adenocarcinoma, GEJ adenocarcinoma, locally advanced esophagogastric adenocarcinoma, relapse free survival, CIBERSORT, immune cell deconvolution

## Abstract

**Background:**

Most patients with resectable locally advanced esophageal and gastroesophageal junction (GEJ) adenocarcinoma (AC) receive concurrent chemoradiation (CRT) followed by esophagectomy. The majority of patients do not achieve pathologic complete response (pCR) with neoadjuvant CRT, and the relapse rate is high among these patients.

**Methods:**

We conducted a phase II study (ClinicalTrials.gov Identifier: NCT02639065) evaluating the efficacy and safety of PD-L1 inhibitor durvalumab in patients with locally advanced esophageal and GEJ AC who have undergone neoadjuvant CRT followed by R0 resection with evidence of persistent residual disease in the surgical specimen. Patients received durvalumab 1500 mg IV every 4 weeks for up to 1 year. The primary endpoint was 1-year relapse free survival (RFS). Secondary endpoint was safety and tolerability of durvalumab following trimodality therapy. Exploratory endpoints included correlation of RFS with PD-L1 expression, HER-2 expression, and tumor immune cell population.

**Results:**

Thirty-seven patients were enrolled. The majority (64.9%) had pathologically positive lymph nodes. The most common treatment related adverse events were fatigue (27%), diarrhea (18.9%), arthralgia (16.2%), nausea (16.2%), pruritus (16.2%), cough (10.8%), and increase in AST/ALT/bilirubin (10.8%). Three (8.1%) patients developed grade 3 immune mediated adverse events. One-year RFS was 73% (95% CI, 56–84%) with median RFS of 21 months (95% CI, 14–40.4 months). Patients with GEJ AC had a trend toward superior 1-year RFS compared to those with esophageal AC (83% vs. 63%, p = 0.1534). There was a numerical trend toward superior 1-year RFS among patients with PD-L1 positive disease compared to those with PD-L1 negative disease, using CPS of ≥10 (100% *vs*. 66.7%, p = 0.1551) and ≥1 (84.2% *vs*. 61.1%, p = 0.1510) cutoffs. A higher relative proportion of M2 macrophages and CD4 memory activated T cells was associated with improved RFS (HR = 0.16; 95% CI, 0.05–0.59; p = 0.0053; and HR = 0.37; 95% CI, 0.15–0.93, p = 0.0351, respectively).

**Conclusions:**

Adjuvant durvalumab in patients with residual disease in the surgical specimen following trimodality therapy for locally advanced esophageal and GEJ AC led to clinically meaningful improvement in 1-year RFS compared to historical control rate. Higher PD-L1 expression may have a correlation with the efficacy of durvalumab in this setting. Higher proportion of M2 macrophages and CD4 memory activated T cells was associated with superior RFS.

## Introduction

Esophageal cancer is the 7^th^ most common cancer and the 6^th^ leading cause of cancer related deaths worldwide (1). While the incidence of squamous cell carcinoma (SCC) of the esophagus has declined in the US, adenocarcinoma (AC) incidence has been rising dramatically (2). Two-thirds of patients with esophageal AC present with locally advanced disease at the time of diagnosis (3). Trimodality therapy with neoadjuvant concurrent chemoradiation (CRT) followed by surgery, as established by the CROSS trial, leads to 5-year overall survival (OS) of 43% in resectable locally advanced esophageal cancer (4). Approximately 23% of patients with AC achieve pathologic complete response (pCR) with neoadjuvant CRT (4). The relapse rate is high in patients who do not achieve pCR and those who have persistent disease in the resected lymph nodes, with 1-year relapse free survival (RFS) of approximately 50% (5–7). Additional post-operative chemotherapy has not been prospectively shown to improve survival in this patient population, and there is a pressing need for novel therapies in this setting (8, 9).

The programmed cell death 1 (PD-1) receptor/programmed cell death ligand 1 (PD-L1) is a well-established immune checkpoint pathway that is exploited by tumors to evade host immune system and has become an attractive target for therapeutic interventions in multiple solid tumors including PD-L1 expressing esophageal AC (10–17). Mounting evidence demonstrates that ionizing radiation, and to a certain extent chemotherapy, may enhance the infiltration of tumor-specific T cells and simultaneously upregulate PD-1/PD-L1 pathway in the tumor microenvironment by inducing DNA damage and promoting immunogenic cell death (18). This upregulation of PD-1/PD-L1 pathway provides a strong scientific rationale for the use of PD-1/PD-L1 inhibitors following CRT, as supported by the efficacy of consolidation durvalumab and pembrolizumab in unresectable stage III non-small cell lung cancer (19, 20).

Based on these data and the activity of immune checkpoint inhibitors (ICIs) in advanced esophageal AC, we designed a phase II trial to evaluate efficacy and safety of PD-L1 inhibitor durvalumab following neoadjuvant CRT and surgery in patients with locally advanced esophageal and gastroesophageal junction (GEJ) AC who had pathologic evidence of residual disease in the surgical specimen.

## Materials and Methods

### Patients

We enrolled patients who were 18 years of age or older and had histologically confirmed locally advanced esophageal or GEJ AC (cTanyN1-3M0 based on AJCC 7^th^ staging system) treated with preoperative CRT followed by R0 resection with histologic evidence of persistent residual disease in the surgical specimen [esophagus/GEJ or lymph node(s) or both]. Eligibility also included an Eastern Cooperative Oncology Group performance status of 0 to 1 and adequate organ function as detailed in the protocol. Key exclusion criteria were presence of active autoimmune disease or any other condition requiring chronic systemic corticosteroids or immunosuppressive agents, a history of primary immunodeficiency, or a history of interstitial lung disease. Acceptable chemotherapy regimens used concurrently with standard dose of radiation included cisplatin and 5-fluorouracil or weekly carboplatin and paclitaxel. Documentation of PD-L1 expression was not required for the enrollment.

### Study Design and Treatment

This was a single arm, multicenter, open label, phase II investigator-initiated trial (ClinicalTrials.gov Identifier: NCT02639065 - Big Ten Cancer Research Consortium study BTCRC-ESO14-012). The primary endpoint was 1-year relapse free survival (RFS) with adjuvant durvalumab. Secondary endpoint was safety and tolerability of durvalumab following trimodality therapy. Exploratory endpoints included correlation of RFS with a variety of biomarkers including PD-L1 expression, HER-2 expression, and tumor immune cell population. Patients received flat dose durvalumab 1500 mg intravenously every 4 weeks, starting within 1 to 3 months following surgery. The treatment was administered for up to 12 months (total of 13 doses) or until unacceptable toxicities, disease relapse, or withdrawal of consent. Dose reductions were not allowed. Dose delays for toxicity were allowed for up to a maximum duration of 42 days. Study schema is shown in **Supplementary Figure S1**.

### Assessments

Patients underwent baseline computed tomography (CT) scan of chest, abdomen, and pelvis within 28 days prior to enrollment on the study and every 3 months during treatment and follow-up for at least 1 year. History and physical examination were performed every 4 weeks during treatment, 30 days following treatment discontinuation, and every 3 months thereafter. Disease relapse was defined as any clinical or radiographic finding(s) that met the criteria for measurable or non-measurable lesions (confirmed by histology/cytology if solitary) according to the Response Evaluation Criteria in Solid Tumors (RECIST) v1.1. RFS was defined as the time from the date of surgery until disease relapse or death from any cause. Patients who remained alive and relapse free were censored at their date of last disease evaluation. One-year RFS was defined as the percentage of patients who were alive and relapse-free at 1 year following surgery. Post-hoc analysis of OS was conducted on available data. OS was defined as time from the date of surgery until death from any cause. OS after relapse was defined as the time from the date of relapse until death from any cause. Patients who remained alive were censored at their last date known alive. Toxicity was evaluated by the collection of adverse events (AEs), serious adverse events (SAEs), and immune related adverse events (irAEs) at every visit and graded according to the National Cancer Institute Common Terminology Criteria for Adverse Events (CTCAE) v4.0. Patients who discontinued durvalumab prior to completion of 1 year of therapy for reasons other than disease relapse were followed every 3 months for 1 year from the time of treatment discontinuation for assessment of disease relapse, survival, and occurrence of any late AEs. Tissue samples from the time of initial diagnosis were collected and banked for correlative analysis.

### PD-L1 and HER-2 Expression Analysis

PD-L1 and HER-2 expressions on the tissue samples obtained at the time of surgery were assessed at Indiana University Pathology Laboratory following completion of the trial. Immunohistochemical (IHC) analysis of PD-L1 expression was performed using 22C3 pharmDx assay on formalin-fixed tumor samples obtained by core-needle biopsy at the time of diagnosis. Expression was categorized according to the combined positive score (CPS) (i.e., the ratio of the combining number of PD-L1 positive tumor cells and immune cells (lymphocytes, macrophages) by IHC staining to the total number of tumor cells) (17). HER-2 expression was analyzed by means of IHC using the HercepTest (Dako) and categorized as negative (IHC 0 or 1+), equivocal (IHC 2+), or positive (IHC 3+) (21).

### Immune Deconvolution Analysis

RNA-sequencing was performed on formalin-fixed paraffin embedded tissue blocks at HudsonAlpha using the Illumuna TruSeq RNA Exome library kit and sequenced on an Illumina NovaSeq 6000. Raw RNA-seq reads were mapped to the human genome (hg38) using the STAR algorithm (22). Read counts for expressed genes were generated using the featureCounts module within the subread package (23). Normalized read counts, fragments per kilobase exon per million mapped reads (FPKM), for each sample were generated using edgeR (24). For immune deconvolution, FPKM values were imported using the web-based tool CIBERSORTx (cibersortx.stanford.edu), which outputs the relative proportion of 22 immune cell types present in each sample (25).

### Study Oversight

The study was designed by the lead investigators at Indiana University and funded by AstraZeneca. The study was conducted in accordance with the principles of the Declaration of Helsinki and Good Clinical Practice guidelines. The protocol was approved by institutional review boards or relevant ethics committees at each of the participating sites. All patients provided written informed consent before screening and enrollment. Clinical data were generated by the investigators and research staff at the participating sites. Safety data were reviewed at regular intervals by study investigators, Indiana University Melvin and Bren Simon Comprehensive Cancer Center’s Data and Safety Monitoring Committee, and the sponsor. All authors had full access to the data, reviewed the manuscript before it was submitted for publication, and provided input. The authors vouch for the accuracy and completeness of the data and analyses. The Big Ten Cancer Research Consortium provided administrative support for the study.

### Statistical Analysis

Statistical analysis was conducted by Biostatistics and Data Management Core at Indiana University Melvin and Bren Simon Comprehensive Cancer Center. Parameter estimates and relevant summary statistics are reported where appropriate. For continuous variables, summary statistics includes number of subjects, mean, median, standard deviation, minimum, and maximum. Categorical endpoints are summarized using number of subjects, frequency, and percentages. Median RFS, OS, and OS after relapse and associated 95% confidence intervals (CI) were estimated using the Kaplan-Meier method. AEs were summarized by treatment relatedness and toxicity grade. Immune deconvolution results were associated with RFS using Cox proportional hazards models with measurements treated as categorical values with optimal cut point defined by the maximal chi-square method. Because of the small sample sizes, the size of the hazard ratio, in addition to the p-value, was to guide interpretation of results. Hazard ratios > 2 (or corresponding less than 0.5) are emphasized. Data analysis was performed in SAS Version 9.4.

We hypothesized that durvalumab will improve the relapse free survival rate at 1 year by 25% compared to historical rate, which would be considered a clinically meaningful improvement. The null hypothesis was that 1-year RFS with adjuvant durvalumab in patients with disease in the surgical specimen following trimodality therapy is 50% or less. The alternative hypothesis was that 1-year RFS with adjuvant durvalumab in this patient population is 75% or greater. With a maximum acceptable type I error of 0.05, and acceptable type II error of 0.20, the calculated sample size was 23 evaluable patients. To improve accuracy for estimating the primary endpoint of 1-year RFS with a 95% CI, an additional 13 patients were planned to be enrolled for at least 34 patients evaluable for 1-year RFS and a target of maximum 39 patients to allow for approximately 10% unevaluable patients. With 34 evaluable patients, if the 1-year RFS is 75%, a 95% two-sided CI will have a half-width of 15% using normal-approximation.

## Results

### Patients and Treatment

Thirty-seven patients were enrolled between April 2016 and September 2019 across three academic sites in the United States. The last patient completed 1 year of follow up in September 2020. Data cutoff for analysis was October 7, 2020. The patient characteristics were consistent with those seen in clinical practice in Western countries with 36 out of 37 patients being male and a median age of 61 years (range, 43–73 years). Eighteen patients (48.6%) had GEJ AC and the remaining had distal esophageal AC. The majority of patients (n = 31, 83.8%) received weekly carboplatin and paclitaxel concurrently with radiation. Nearly two-thirds of patients (n = 24, 64.9%) had pathologically positive lymph nodes in the surgical specimen. The baseline characteristics are summarized in **Table 1**.

**Table 1:** Baseline patient and disease characteristics.

Characteristic	Value
**Patients enrolled**	37
**Sex**	
Female	1
Male	36
**Age, y**	
Median (range)	61 (43–73)
**Enrolling site**	
Indiana University	25
University of Michigan	7
University of Iowa	5
**Site of disease**	
GEJ	18
Distal Esophagus	19
**Chemotherapy regimen**	
Cisplatin + 5-fluorouracil	6
Carboplatin + paclitaxel	31
**Pathologic lymph node stage**	
N3	3
N2	10
N1	11
N0	13
T3	8
T2	2
T1	2
T0	1

Median time from surgery to initiation of durvalumab treatment was 2.4 months (95% CI = 2.1–2.6 months). The median number of doses received was 12 (range, 1–13). Seventeen (45.9%) patients completed 1 year of durvalumab. The reasons for treatment discontinuation in the remaining 20 patients were disease relapse (n = 11, 29.7%), AEs (n = 8, 21.6%), and consent withdrawal (n = 1, 2.7%).

### Efficacy

**Figure 1:**
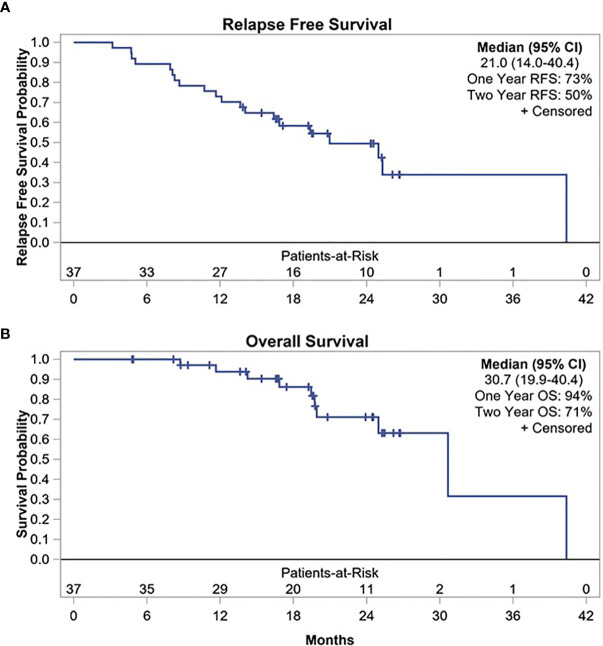
**(A)** Relapse free survival, **(B)** Overall survival with durvalumab.

At median follow-up time of 17.7 months (range, 1.7–24.3 months), 20 patients experienced disease relapse. Of these, 10 relapses occurred within the first year after surgery with 1-year RFS of 73% (95% CI, 56–84%) **(** **Figure 1** **)**. Two-year RFS was 50% (95% CI, 33–66%) with median RFS of 21 months (95% CI, 14–40.4 months). In addition, when restricting the primary endpoint analysis to the original planned sample size of 23, the estimate of 1-year RFS was 78% (95% CI, 55–90%), so the previously planned endpoint was met **(** **Supplementary Figure S2** **)**. Patients with GEJ AC had a trend toward superior 1-year RFS compared to those with esophageal AC (83% *vs*. 63%, p = 0.1534). Similarly, median RFS was longer among patients with GEJ AC compared to esophageal AC which did not reach statistical significance (25 months *vs*. 16.8 months, p = 0.4282) **(** **Supplementary Figure S3** **)**.

**Figure 2:**
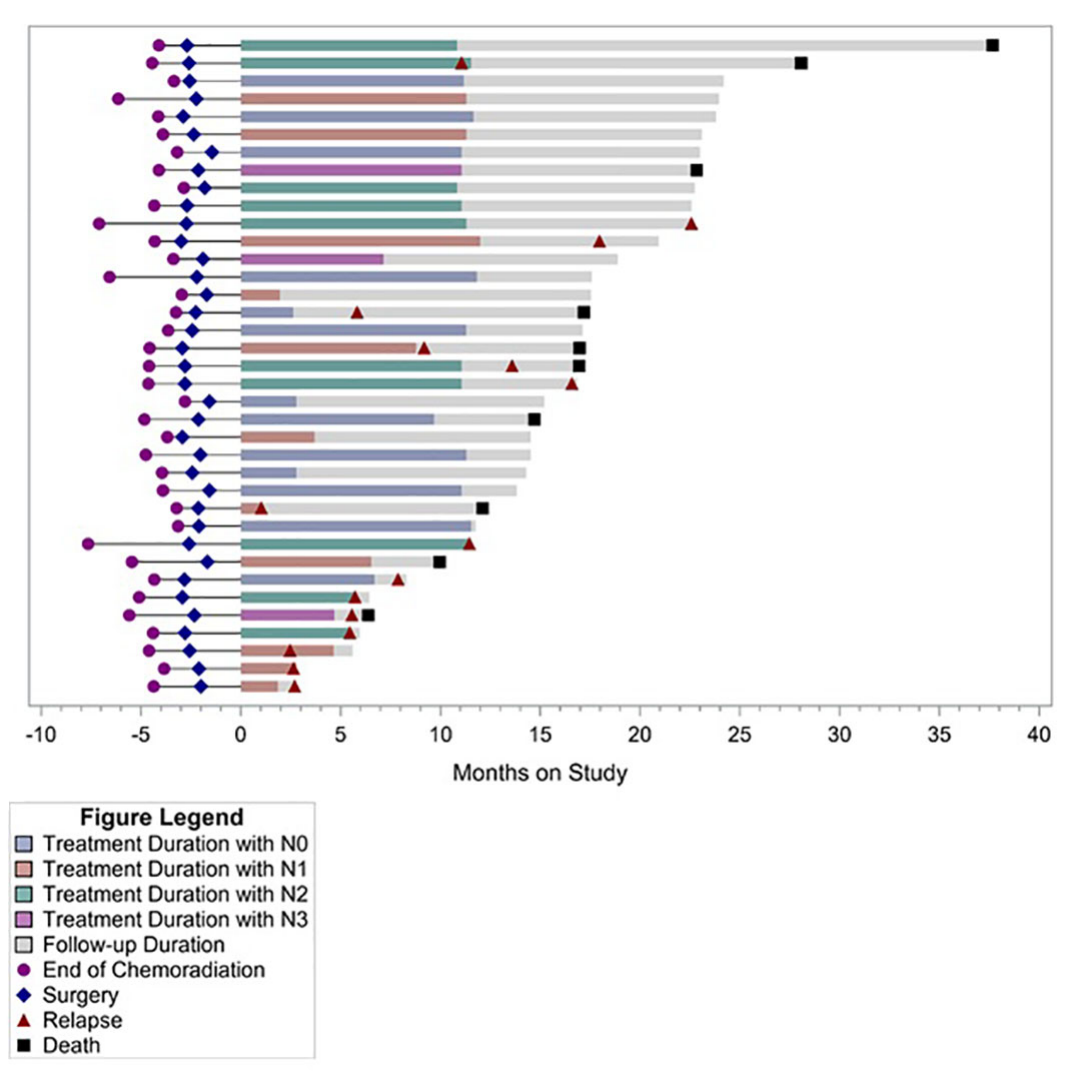
Treatment duration, relapse, and follow-up based on pathologic lymph node status.

Post-hoc analysis of OS showed 1-year OS of 94%, 2-year OS of 71%, and median OS of 30.7 months (95% CI, 19.9–40.4 months) (**Figure 1** **)**. Median OS after relapse was 11.1 months (95% CI, 0.8–17 months). All 10 relapses within the first year after surgery were systemic relapses. However, three of the later relapses were locoregional which were treated with repeat CRT. None of the patients received an ICI as a part of subsequent therapy for recurrent disease. Longer term follow-up data were available on 19 (51%) patients. Of these, five did not experience disease relapse while two experienced locoregional relapse and received repeat CRT. All of these seven patients were alive and disease free at median of 47.8 months and 40 months following surgery and discontinuation of durvalumab, respectively. Five of these patients had positive lymph nodes at the time of surgery. **Figure 2** summarizes the duration of treatment, disease relapse, and death based on pathologic lymph node stage.

### Safety

Most patients (n = 30, 81.1%) experienced at least one AE of any grade. The most common treatment related AEs occurring in ≥10% of patients were fatigue (27%, n = 10), diarrhea (18.9%, n = 7), arthralgia (16.2%, n = 6), nausea (16.2%, n = 6), pruritus (16.2%, n = 6), cough (10.8%, n = 4), and increase in AST/ALT/bilirubin (10.8%, n = 4) **(** **Table 2** **)**. Ten (27%) patients experienced grade 3 AEs which were initially considered to be at least possibly related to durvalumab. Of these, three (hematuria, hypoglycemia, and decreased platelet count) were later determined not to be treatment related and these patients completed 1 year of durvalumab. Two AEs (encephalopathy and increase in AST) were later attributed to disease progression rather than durvalumab. One patient with grade 3 elevation of AST, ALT, and CPK was found to have severe hypothyroidism. The laboratory abnormalities resolved with thyroid replacement therapy, and the patient eventually completed 1 year of durvalumab. One patient developed grade 3 sick sinus syndrome and was taken off therapy. The remaining 3 patients developed grade 3 immune related adverse events (irAEs), including pneumonitis (n = 1), hepatitis (n = 1), and colitis (n = 1), that required treatment discontinuation. Two of these three patients were alive and disease free at 48 and 36 months from discontinuation of therapy, respectively. Four additional patients discontinued treatment because of grade 2 colitis, failure to thrive, creatinine elevation, and recurrent grade 2 AEs. No treatment-related deaths were observed.

**Table 2:** Treatment related adverse events occurring in ≥10% of patients and possible immune-related adverse events.

Adverse event	Any grade, no. (%)	Grade 1–2, no. (%)	Grade 3, no. (%)	Grade ≥4, no. (%)

	10 (27%)	10 (27%)	0 (0.0)	0 (0.0)
Diarrhea	7 (18.9%)	6 (16.2%)	1 (2.7%)	0 (0.0)
Arthralgia	6 (16.2%)	6 (16.2%)	0 (0.0)	0 (0.0)
Nausea	6 (16.2%)	6 (16.2%)	0 (0.0)	0 (0.0)
Pruritus	6 (16.2%)	6 (16.2%)	0 (0.0)	0 (0.0)
Cough	4 (10.8%)	3 (8.1%)	1 (2.7%)	0 (0.0)
**Possible immune-related adverse event**				
Diarrhea	7 (18.9%)	6 (16.2%)	1 (2.7%)	0 (0.0)
Elevated AST	4 (10.8%)	1 (2.7%)	3 (8.1%)	0 (0.0)
Elevated ALT	4 (10.8%)	3 (8.1%)	1 (2.7%)	0 (0.0)
Elevated Bilirubin	4 (10.8%)	3 (8.1%)	1 (2.7%)	0 (0.0)
Hyperthyroidism	3 (8.1%)	3 (8.1%)	0 (0.0)	0 (0.0)
Colitis	2 (5.4%)	2 (5.4%)	0 (0.0)	0 (0.0)
Skin Rash	2 (5.4%)	2 (5.4%)	0 (0.0)	0 (0.0)
Adrenal Insufficiency	1 (2.7%)	1 (2.7%)	0 (0.0)	0 (0.0)
Hypothyroidism	1 (2.7%)	1 (2.7%)	0 (0.0)	0 (0.0)
Pneumonitis	1 (2.7%)	0 (0.0)	1 (2.7%)	0 (0.0)

AST, aspartate aminotransferase; ALT, alanine aminotransferase.

### Exploratory Analysis

**Figure 3:**
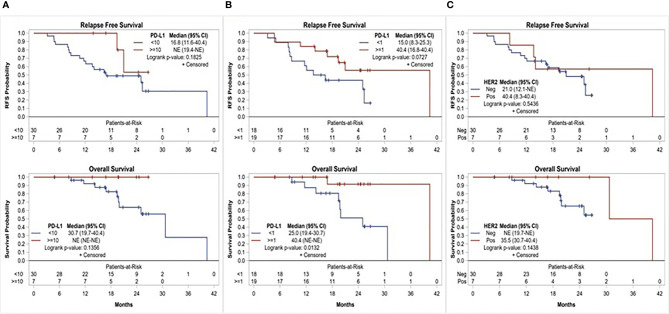
Relapse free survival and overall survival based on, **(A)** PD-L1 expression using CPS≥10 cutoff, **(B)** PD-L1 expression using CPS≥1 cutoff, and **(C)** HER-2 status.

Of the 37 patients, 19 (51.4%) had PD-L1 CPS ≥1 and 7 (18.9%) had PD-L1 CPS ≥10. There was a numerical, yet statistically nonsignificant, trend toward superior 1-year RFS among patients with PD-L1 positive disease compared to those with PD-L1 negative disease, using CPS ≥10 (100% *vs*. 66.7%, p = 0.1551) and ≥1 (84.2% *vs*. 61.1%, p = 0.1510) cutoffs. Similarly, median RFS and OS were numerically superior among patients with PD-L1 CPS ≥10 compared to <10 (median RFS: not reached *vs*. 16.8 months, p = 0.1825; and median OS: not reached *vs*. 30.7 months, p = 0.1356). Using CPS ≥1 as a cutoff, patients with PD-L1 positive disease had numerically superior RFS (median RFS: 40.4 *vs*. 15 months, p = 0.0727) and superior OS that was clinically as well as statistically significant (median OS: 40.4 vs. 25.0 months, p = 0.0132). Overall, 7(18.9%) patients had HER-2 positive disease. Compared to patients with HER-2 negative disease, those with HER-2 positive disease had numerically superior 1-year RFS (85.7% *vs*. 70%, p = 0.6471) and median RFS (40.4 *vs*. 21 months, p = 0.5436); however, this trend was not statistically significant (**Figure 3**).

### CIBERSORT

**Figure 4:**
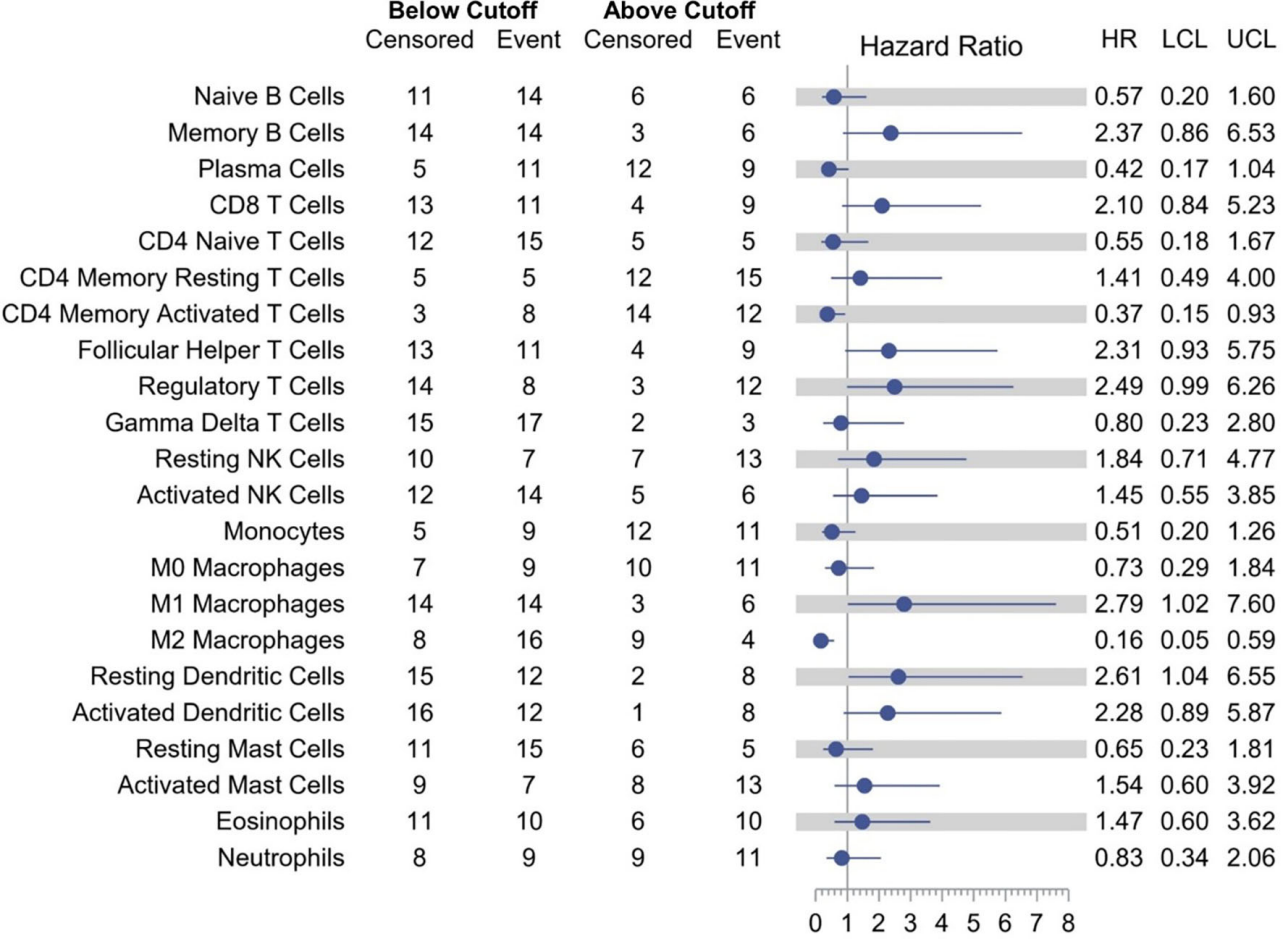
Association of tumor immune cell population with RFS.

Models examining the association between RFS and tumor immune cell population are presented in **Table 3**. A relative proportion of M1 tumor associated macrophage (TAM) greater than 0.0205 was associated with a 179% increase in the hazard of relapse or death compared to a relative proportion of less than or equal to 0.0205 (HR = 2.79; 95% CI, 1.02–7.60; p = 0.0448). Similarly, a relative proportion of resting dendritic cells greater than 0.0303 was associated with a 161% increase in the hazard of relapse or death compared to a relative proportion of less than or equal to 0.0303 (HR = 2.61; 95% CI, 1.04–6.55; p = 0.0402). On the other hand, a relative proportion of CD4 memory activated T cells greater than 0.0306 was associated with a 63% decrease in the hazard of relapse or death compared to a relative proportion of less than or equal to 0.0306 (HR = 0.37; 95% CI, 0.15–0.93; p = 0.0351), and a relative proportion of M2 TAM greater than 0.0864 was associated with a 84% decrease in the hazard of relapse or death compared to a relative proportion of less than or equal to 0.0864 (HR = 0.16; 95% CI, 0.05–0.59; p = 0.0053) (**Figure 4**). Other cell types that were not significant at the p = 0.05 level but with large (>2.0 or <0.5) HRs for RFS were memory B cells (HR = 2.37), CD8 T cells (HR = 2.10), follicular helper T cells (HR = 2.31), regulatory T cells (HR = 2.49), activated dendritic cells (HR = 2.28), and plasma cells (HR = 0.42).

**Table 3:** Cox proportional hazards models for CIBERSORT data.

Cell type	HR	95% confidence interval	p-value
**Naïve B cells,** >0.0213 *vs*. <=0.0213	0.57	0.20–1.60	0.2855
**Memory B cells,** >0.0063 *vs*. <=0.0063	**2.37**	**0.86**–**6.53**	**0.0950**
**Plasma cells,** >0.0345 *vs*. <=0.0345	**0.42**	**0.17**–**1.04**	**0.0616**
**CD8 T cells,** >0.0215 *vs*. <=0.0215	**2.10**	**0.84**–**5.23**	**0.1110**
**CD4 naïve T cells,** >0 *vs*. <=0	0.55	0.18–1.67	0.2945
**CD4 memory resting T cells,** >0.1389 *vs*. <=0.1389	1.41	0.49–4.00	0.5232
**CD4 memory activated T cells,** >0.0306 *vs*. <=0.0306	**0.37**	**0.15-0.93**	**0.0351**
**Follicular helper T cells,** >0.0378 *vs*. <=0.0378	**2.31**	**0.93**–**5.75**	**0.0718**
**Regulatory T cells,** >0.0147 *vs*. <=0.0147	**2.49**	**0.99**–**6.26**	**0.0524**
**Gamma delta T cells,** >0 *vs*. <=0	0.80	0.23–2.80	0.7309
**Resting NK cells,** >0.0092 *vs*. <=0.0092	1.84	0.71–4.77	0.2073
**Activated NK cells,** >0.0509 *vs*. <=0.0509	1.45	0.55–3.85	0.4514
**Monocytes,** >0.0137 *vs*. <=0.0137	0.51	0.20–1.26	0.1421
**Macrophages M0,** >0.1340 *vs*. <=0.1340	0.73	0.29–1.84	0.5064
**Macrophages M1,** >0.0205 *vs*. <=0.0205	**2.79**	**1.02**–**7.60**	**0.0448**
**Macrophages M2,** >0.0864 *vs*. <=0.0864	**0.16**	**0.05**–**0.59**	**0.0053**
**Resting dendritic cells,** >0.0303 *vs*. <=0.0303	**2.61**	**1.04**–**6.55**	**0.0402**
**Activated dendritic cells,** >0.0268 *vs*. <=0.0268	**2.28**	**0.89**–**5.87**	**0.0878**
**Resting mast cells,** >0.1146 *vs*. <=0.1146	0.65	0.23–1.81	0.4097
**Activated mast cells,** >0.0069 *vs*. <=0.0069	1.54	0.60–3.92	0.3695
**Eosinophils,** >0.0420 *vs*. <=0.0420	1.47	0.60–3.62	0.4036
**Neutrophils,** >0.0127 *vs*. <=0.0127	0.83	0.34–2.06	0.6933

The bold values are for the cell types with large (>2.0 or <0.5) HRs for RFS.

## Discussion

Most patients with resectable locally advanced esophageal and GEJ AC receive concurrent CRT followed by surgical resection (4). As opposed to esophageal SCC, the majority of patients with AC do not achieve pCR following neoadjuvant CRT (4). These patients carry poor prognosis with a high risk of disease relapse within the first year following curative intent trimodality therapy. The upregulation of PD-1/PD-L1 pathway induced by radiation and possibly chemotherapy presents a unique opportunity to use immune checkpoint inhibition in this setting. The results of our study indicate that adjuvant therapy with PD-L1 inhibitor durvalumab in patients who do not achieve pCR following trimodality therapy leads to improvement in 1-year RFS compared to historical control rate. The study reached its hypothesized primary endpoint in the initially planned cohort of 23 patients with 1-year RFS of 78%. While the 1-year RFS in the entire cohort of 37 patients was 73%, this improvement is still clinically meaningful since the majority of patients had pathologically positive lymph nodes, including 13 (35%) patients with pN2 or pN3 disease who are at the highest risk of developing systemic disease recurrences shortly after surgery and have median OS of less than 10 months (5).

Adjuvant studies with ICIs are fraught with the notion that the microscopic burden of disease associated with minimal neoantigen load may lead to suboptimal efficacy of an ICI. This did not appear to be the case in our study and in recently reported randomized CheckMate-577 trial comparing nivolumab with placebo following trimodality therapy for similar patient population showing superior disease free survival (DFS) with nivolumab (median DFS, 22.4 *vs*. 11 months, HR 0.69; 95% CI, 0.56–0.86, p < 0.001) (26). With the caveat of cross trial comparison, our results are in alignment with CheckMate-577 trial leading to FDA approval of nivolumab in this setting. The median RFS of 21 months (95% CI, 14–40.4 months) in our trial is similar to the reported median DFS of 22.4 months in the nivolumab arm in CheckMate-577 trial, indicating that PD-L1 inhibitor durvalumab may have a similar efficacy as PD-1 inhibitor nivolumab in this setting. In contrast to CheckMate-577 which included nearly 30% of patients with esophageal SCC, our study was restricted to patients with adenocarcinoma histology. It is relevant to outline this distinction given the fundamental genomic differences between SCC and AC, and the historically greater sensitivity of SCC to ICIs (15, 27–29). Another intriguing contrast between the two studies is the relapse- or disease-free survival with ICI in patients with GEJ AC. The subset analysis of CheckMate-577 study showed that the HR of median DFS was inferior and the CI crossed the boundary of statistical non-significance in patients with GEJ tumors (HR 0.87; 95% CI, 0.63–1.21) compared to those with esophageal tumors (HR 0.61; 95% CI, 0.47–0.78). In contrast, patients with GEJ AC seemed to fare better than those with esophageal AC in our study, albeit the difference was not statistically significant because of small sample size. While none of these studies were specifically designed to answer this question and the data are derived from post-hoc subset analyses, this difference is hypothesis generating and calls for further research into the immune repertoire of upper GI AC originating from different locations and statistically powered prospective comparison of efficacy of ICI between GEJ AC and esophageal AC.

From a safety perspective, it was feasible to initiate therapy with durvalumab within 3 months of esophagectomy, a surgery that is typically associated with significant post-operative morbidity. However, only 46% (n = 17) of patients were able to complete the intended 12 months of therapy which is comparable to 43% treatment completion rate reported in CheckMate-577 trial and PACIFIC trial of durvalumab following concurrent CRT in unresectable stage III non-small cell lung cancer (19). Similarly, in the MAGIC trial of perioperative chemotherapy and surgery versus surgery alone which included approximately 25% of patients with distal esophageal or GEJ AC, only 55% of patients who were assigned to receive perioperative chemotherapy ended up receiving any postoperative chemotherapy (30). This finding raises a question of whether administration of ICI in a pre-operative setting, especially concurrently with CRT, may offer a greater benefit by obtaining maximum advantage of the synergy of ICI with CRT and the ability to deliver the desired amount of drug in the pre-operative setting. Eight (21.6%) patients discontinued durvalumab due to AEs, which is slightly higher than 15.4% reported in PACIFIC trial and 14% in CheckMate-577 trial. Two of the three patients who experienced irAE leading to early discontinuation of durvalumab had significantly longer RFS, corroborating with the emerging body of evidence suggesting greater efficacy of ICIs in patients who develop irAE (31).

Clinical trials utilizing ICIs in metastatic gastroesophageal AC have shown positive correlation between therapeutic efficacy of immunotherapy and PD-L1 expression (17, 29, 32). However, a well-defined cutoff for PD-L1 expression and other biomarkers of response to immunotherapy to guide optimum patient selection are lacking. Our study showed a trend toward improved RFS and OS in patients with PD-L1 positive disease. While both traditionally used PD-L1 expression cutoffs to define positivity, CPS ≥1 and ≥10, seemed to correlate with efficacy of durvalumab in post-trimodality setting, the trend was more pronounced among patients with PD-L1 CPS ≥10 disease, with all seven patients being relapse free at 1 year. Except for the difference in median OS among patients with PD-L1 CPS≥1 versus <1, none of the other comparisons were statistically significant. This is at least in part explained by very small sample size in each subgroup. Moreover, retrospective evaluation of PD-L1 expression by IHC in banked tissue samples could have been affected by the duration and mode of tissue storage as described in the literature (33–35). Nevertheless, these findings are hypothesis generating and may imply that PD-L1 expression is a relevant biomarker of the efficacy of adjuvant durvalumab following trimodality therapy in locally advanced disease. There was a trend toward improved 1-year RFS in HER-2 positive disease with durvalumab, which was limited by a small sample size. With the emerging data showing synergistic activity between PD-1/PD-L1 inhibitors and HER-2 targeting treatments in metastatic gastroesophageal cancer, a combination strategy in the adjuvant setting will be intriguing (36–38). Further exploratory analysis involving immune deconvolution demonstrated that relative proportion of M2 TAM higher than the optimum cutoff was associated with improved RFS with durvalumab. A higher infiltration of M2 TAMs has been associated with aggressive tumor biology, promotion of angiogenesis, chemoresistance, and poor prognosis in multiple tumor types (39–41). A possible explanation for the improved RFS in patients with higher proportion of M2 TAMs in our study is that M2 TAMs are associated with high PD-L1 expression on tumor cells and immune cells, and may have contributed to improved efficacy of PD-L1 inhibitor (42). Similarly, a higher proportion of memory activated CD4 T cells was associated with improved RFS, possibly from facilitation of anti-tumor response from cytotoxic T cells, production of effector cytokines such as IFNγ and tumor necrosis factor-α (TNFα), and induction of B cell driven humoral responses against tumor antigens (43). On the other hand, an increase in proportion of resting dendritic cells was associated with worse RFS, possibly explained by induction of peripheral CD8 T cell tolerance (44). Intriguingly, a higher proportion of M1 TAM was associated with inferior RFS with durvalumab, which is contrary to what would be expected given their role in release of pro-inflammatory cytokines and induction of anti-tumor immune response. As the immune cell population analysis is limited by small sample size, the results are hypothesis generating and need to be explored further in a larger patient population.

Our study is limited by its small sample size and non-randomized design. The historical control rate for 1-year RFS of patients who did not obtain pCR with neoadjuvant CRT was largely derived from retrospective studies as this is not reported in prospective trials (6, 7). Additionally, it is debatable whether the endpoint of 1-year RFS with an adjuvant therapy given for 1 year is an acceptable surrogate endpoint of long-term benefit. It remains unclear if durvalumab in the adjuvant setting is merely delaying the disease relapse or eliminating micrometastatic disease. Nevertheless, longer term follow-up data indicate that a subset of patients (n = 7) did derive durable benefit from durvalumab and have remained relapse free for nearly 3.5 years following discontinuation of therapy. Interestingly, while many relapses in our study were systemic, a small subset of patients who experienced late relapses following discontinuation of durvalumab had locoregional disease that was amenable to curative intent therapy. The OS results from CheckMate-577 may elucidate the effect of post-relapse therapies on OS in the post-ICI approval era. Finally, the biomarker analysis was largely exploratory and microsatellite instability (MSI) status was not available for all patients.

In conclusion, adjuvant durvalumab in patients with residual disease in the surgical specimen following neoadjuvant CRT and R0 resection for locally advanced esophageal and GEJ AC led to a clinically meaningful improvement in 1-year RFS compared to historical control rate. The safety profile of durvalumab was consistent with what has been previously reported; however, less than 50% of patients were able to complete intended duration of therapy. Higher PD-L1 expression may have a correlation with the efficacy of durvalumab in this setting and needs to be confirmed prospectively. Relatively higher proportion of M2 TAMs and CD4 memory activated T cells was associated with improved RFS with durvalumab, which needs to be explored further.

## Data Availability Statement

The original contributions presented in the study are included in the article/**Supplementary Material**. Further inquiries can be directed to the corresponding author. The RNA-seq data presented in the study are deposited in the NCBI GEO repository (https://www.ncbi.nlm.nih.gov/geo/query/acc.cgi?acc=GSE183924), accession number GSE183924.

## Ethics Statement

The studies involving human participants were reviewed and approved by institutional review boards or relevant ethics committees at each of the participating sites. The patients/participants provided their written informed consent to participate in this study.

## Author Contributions

All authors have contributed sufficiently to the generation of data, analysis, and preparation of the manuscript. All authors contributed to the article and approved the submitted version.

## Funding

The study was funded by AstraZeneca. Correlative studies, including analysis of PD-L1 and HER-2 expression, were performed utilizing funding from George and Sarah Jane Fisher Young Investigator Award received by Mamdani H. from Hoosier Cancer Research Network. The funder was not involved in the study design, collection, analysis, and interpretation of data, the writing of this article or the decision to submit it for publication.

## Conflict of Interest

The authors report following relationships with the companies, none of which has resulted in an actual or potential conflict of interest with regard to this manuscript. HM: Consulting/Advisory board – AstraZeneca, Zentalis; Travel Accommodations: AstraZeneca (IITpresentation at Immuno-oncology conference). BS: Clinical trials funding to the institution – Merck. P. Kasi: Consultancy/advisory board: Taiho (to institution), Ipsen (to institution), Foundation Medicine, Bayer, Axiom, Natera, Roche, IPBA, Merck, QED, Tempus Labs, Daiiche Sankyo, Boston Health Care, Delcath, Eli Lily; Research/Grant Funding: BMS (institution), Advanced Accelerator Applications (institution), Array Biopharma (institution), Tesaro (institution), Boston Scientific (institution), Celgene (institution); Travel Accommodations: AstraZeneca (IITpresentation at Immuno-oncology conference). SJ: Consulting/Advisory board – Adaptimmune; Clinical trials funding to the institution- AstraZeneca, Tesaro, Astex.

The remaining authors declare that the research was conducted in the absence of any commercial or financial relationships that could be construed as a potential conflict of interest.

## Publisher’s Note

All claims expressed in this article are solely those of the authors and do not necessarily represent those of their affiliated organizations, or those of the publisher, the editors and the reviewers. Any product that may be evaluated in this article, or claim that may be made by its manufacturer, is not guaranteed or endorsed by the publisher.
