# Supplementary material for: A Phase II Trial of Adjuvant Durvalumab Following Trimodality Therapy for Locally Advanced Esophageal and Gastroesophageal Junction Adenocarcinoma: A Big Ten Cancer Research Consortium Study
Source: Front Oncol. 2021 Sep 17;11:736620. doi: 10.3389/fonc.2021.736620 (PMC8484871; doi:10.3389/fonc.2021.736620)

**Supplementary Figure 1: Study Schema**

Durvalumab 1500 mg

Every 4 weeks

Up to 12 months (13 doses)

No disease recurrence

Disease Assessment every 3 months

Significant toxicity or withdrawal of consent

Continue treatment up to 12 months (13 doses)

Discontinue treatment

Patients with locally advanced esophageal cancer without distant metastasis who have received neoadjuvant concurrent chemoradiation followed by surgery and have persistent residual disease in the surgical sample (esophagus or lymph node or both), including patients with marked (<10% residual tumor), moderate (10-50% residual tumor) or no definite response (>50% residual tumor).

Follow for relapse free survival for 1 year after treatment discontinuation.

Disease recurrence

Discontinue treatment

**Supplementary Figure 2: A. Relapse free survival, B. Overall survival with durvalumab in first 23 evaluable patients**


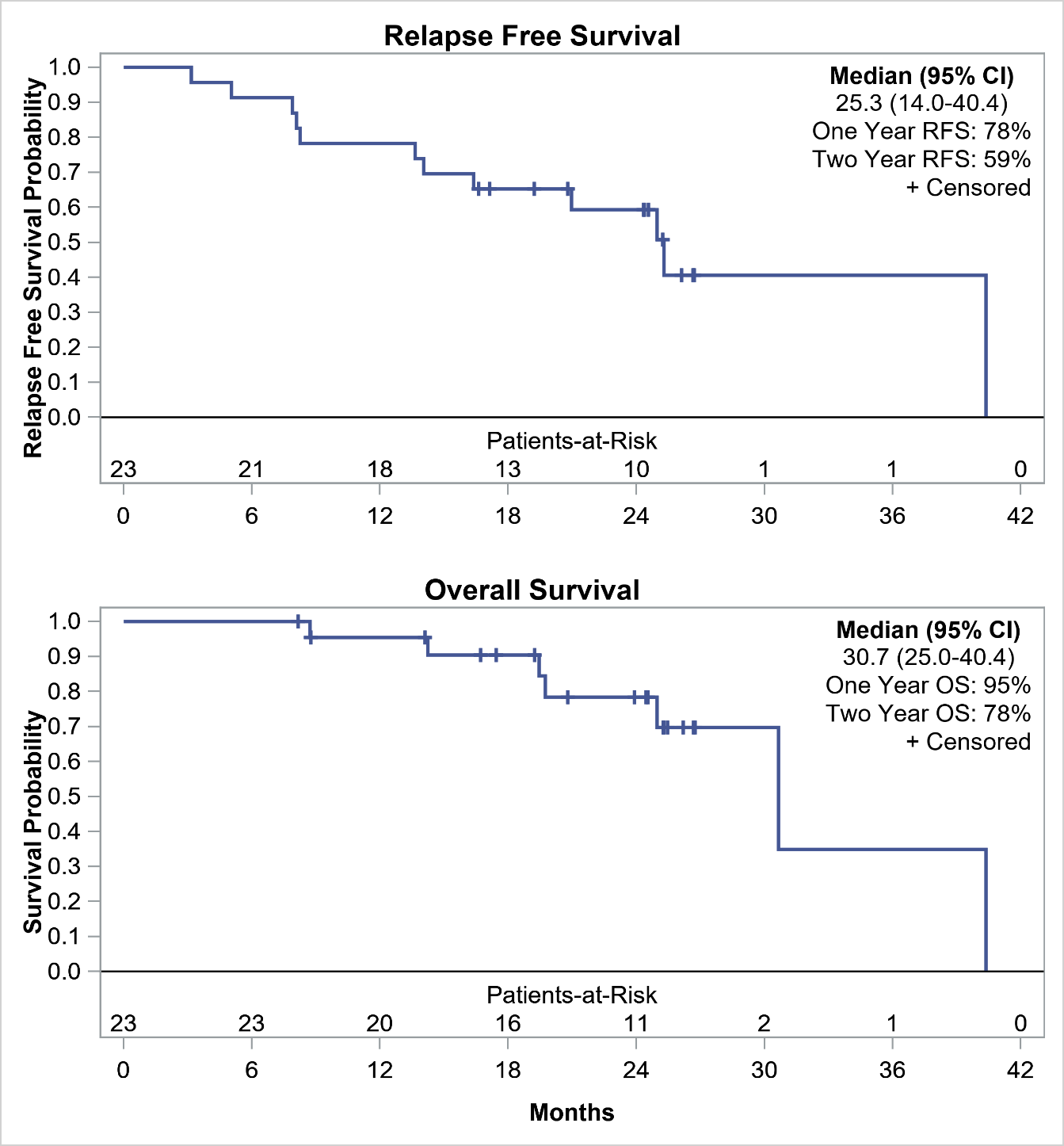


**B**

**A**

**Supplementary Figure 3: Relapse free survival by tumor location**


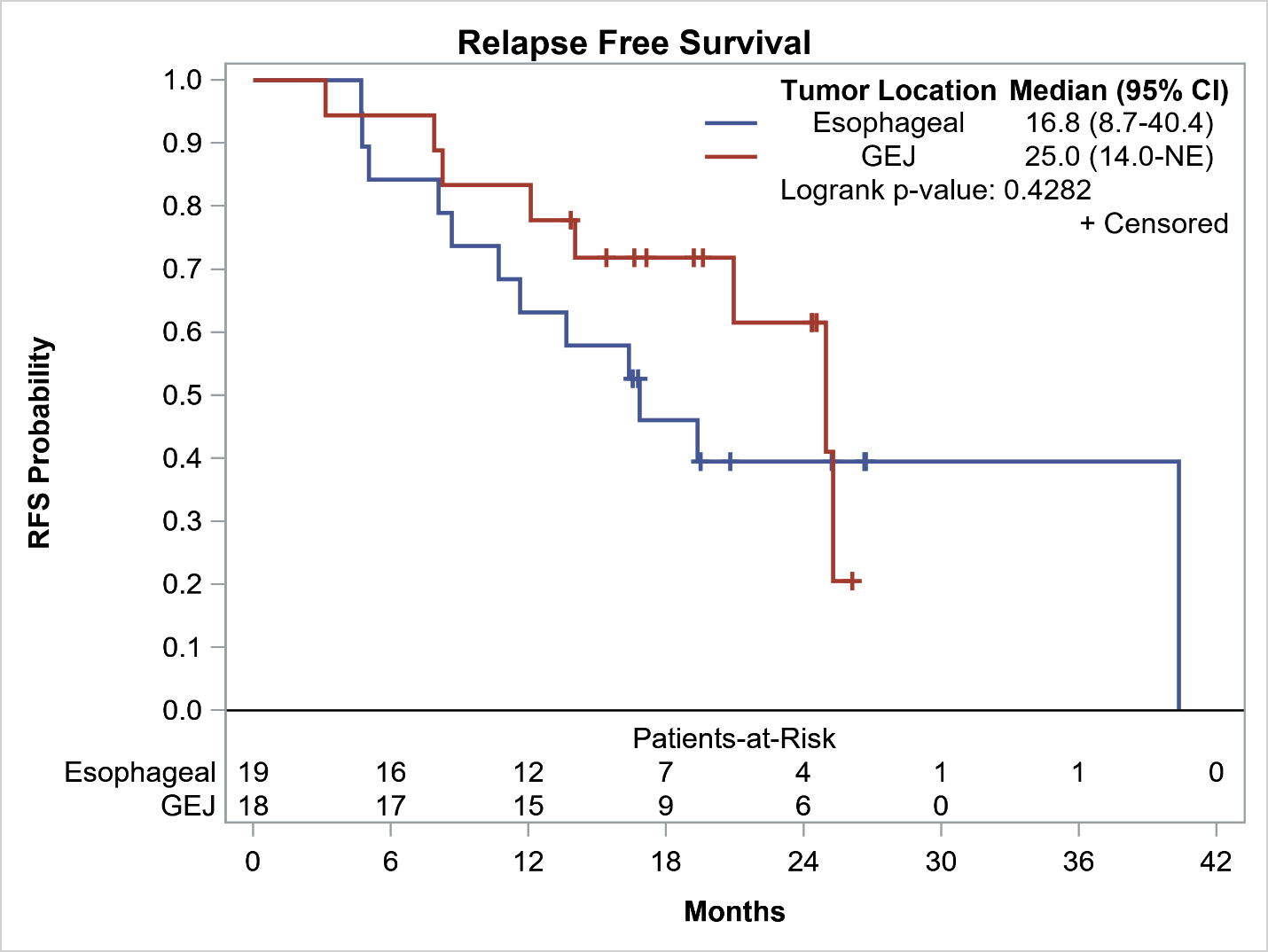

Supplement: Supplementary file 1 [file DataSheet_1.docx]
